# Emerging Roles of RNF168 in Tumor Progression

**DOI:** 10.3390/molecules28031417

**Published:** 2023-02-02

**Authors:** Tianyuan Xie, Hai Qin, Zhengdong Yuan, Yiwen Zhang, Xiaoman Li, Lufeng Zheng

**Affiliations:** 1 Jiangsu Key Laboratory of Carcinogenesis and Intervention, School of Life Science and Technology, China Pharmaceutical University, 639 Longmian Road, Nanjing 211198, China; 2 Department of Clinical Laboratory, Guizhou Provincial Orthopedic Hospital, No. 206, Sixian Street, Baiyun District, Guiyang 550007, China; 3 Jiangsu Key Laboratory for Pharmacology and Safety Evaluation of Chinese Materia Medica, School of Pharmacy, Nanjing University of Chinese Medicine, Nanjing 210023, China

**Keywords:** RING finger protein family, RNF168, DNA damage repair, cancer, E3 ubiquitin ligase

## Abstract

RING finger protein 168 (RNF168) is an E3 ubiquitin ligase with the RING finger domain. It is an important protein contributing to the DNA double-strand damage repair pathway. Recent studies have found that RNF168 is significantly implicated in the occurrence and development of various cancers. Additionally, RNF168 contributes to the drug resistance of tumor cells by enhancing their DNA repair ability or regulating the degradation of target proteins. This paper summarizes and prospects the research progress of the structure and main functions of RNF168, especially its roles and the underlying mechanisms in tumorigenesis.

## 1. Introduction

Post-protein ubiquitination is a ubiquitous form of post-translational modification (PTM), which occurs through a three-enzyme cascade (ubiquitin-activating enzyme 1 (E1), ubiquitin-activating enzyme 2 (E2), ubiquitin ligase 3 (E3)). E3 ligase is involved in the last step of the ubiquitination cascade by specifically binding to substrate proteins, subsequently guiding ubiquitin in binding to the lysine residues of target proteins and thus regulating many cellular processes, such as protein degradation, DNA repair, and signal transduction [1]. Currently, E3 ligases attract increasing attention because they can regulate the stability and function of proteins, based on which some methods or targets have been explored for anti-tumor drug development, such as the Proteolysis-Targeting Chimeras (PROTAC) technology [2,3].

RNF168 belongs to RING E3s and has been widely studied, especially its roles in DNA repair pathways [4]. Additionally, the abnormal expression of RNF168 has been identified in many cancers [5]. In this review, we summarized the current studies on the structure and function of RNF168. In addition, we also discussed the potential mechanism underlying RNF168 effects on tumorigenesis and progression. However, the research on the clinical significance of RNF168 and its role in cancer proliferation or inhibition is still elusive. Here, we hope to clarify the great potential for targeting RNF168 as a cancer treatment target.

## 2. RING Finger Protein Family

**Figure 1:**
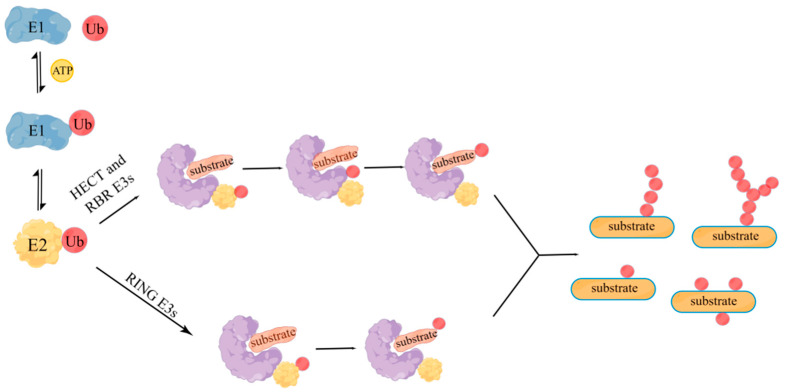
Ubiquitination mediated by three ubiquitin ligase families. Ubiquitination modification involves a series of reactions of ubiquitin-activating enzyme E1, ubiquitin-binding enzyme E2, and ubiquitin ligase E3: firstly, when ATP is supplied, E1 enzyme adheres to the tail of ubiquitin molecule, and then E1 enzyme transfers the activated ubiquitin molecule to E2 enzyme. Then, E2 enzyme and some different E3 enzymes jointly recognize the target protein and modify it. The E3 enzyme is shaped like a clip, and the target protein is connected in the middle gap. The left domain of the enzyme determines the specific recognition of the target protein, and the right domain locates the E2 enzyme to transfer ubiquitin molecules. According to the ubiquitin transfer mechanism of E3 enzymes, E3 enzymes are divided into RING, HECT, and RBR families. HECT and RBR E3s contain a catalytic cysteine that receives ubiquitin from E2 ubiquitin complex, and then transfer the ubiquitin to substrate; while RING E3s can directly catalyze the transfer of ubiquitin from E2 ubiquitin complex to substrate.

PTM is an important step in protein biosynthesis and has a great impact on protein function. Ubiquitination is a kind of PTM, similar to other PTM systems such as methylation, acetylation, and phosphorylation, protein ubiquitination has become an important process for a series of biological activities [6]. Dysfunction of the ubiquitination system can lead to various diseases, such as cancer and neurodegenerative diseases [7,8]. According to the catalytic domain, E3 is usually categorized as three families: the RING domain family, HECT (homologous to E6AP C-terminals), and RBR (RING between RING) [9], all of which have two main ubiquitin transfer mechanisms. HECT and RBR E3s contain a catalytic cysteine that receives ubiquitin from E2 ubiquitin complex, and then transfer the ubiquitin to substrate; while RING E3s can directly catalyze the transfer of ubiquitin from E2 ubiquitin complex to substrates [10]. RING E3s have attracted increasing attention because of their mediated-atypical ubiquitination mechanism and unique regulatory function (Figure 1).

**Figure 2:**
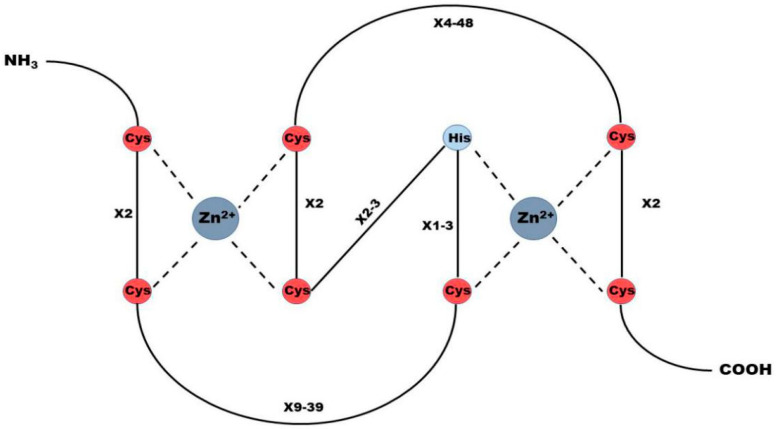
Structure of RING E3 ubiquitin ligase. RING finger motif is an essential factor to ensure that RING E3s has the function of ubiquitin ligase. It is composed of 40–60 residues, of which 8 spatially conserved amino acid residues are arranged in the order of Cys-X2-Cys-X(9–39)-Cys-X(1–3)-His-X(2–3)-Cys-X2-Cys-X(4–48)-Cys-X2-Cys (X represents any residue, His and Cys can be exchanged). The domain formed by this residue can chelate two zinc ions and provide a binding platform for E2 enzyme.

Among the E3 families, RING E3s is the largest E3 family including more than 600 proteins encoded by mammalian genome, which are characterized as one or two RING finger motifs and a “cross scaffold” topology that coordinates two zinc ions [11,12]. RING finger motifs are essential for the function of RING E3s, and are composed of 40–60 residues, in which eight spatially conserved amino acid residues are arranged in the order of Cys-X2-Cys-X(9–39)-Cys-X(1–3)-His-X(2–3)-Cys-X2-Cys-X(4–48)-Cys-X2-Cys (X represents any residue, His and Cys can be exchanged). The domain formed by these residues can chelate two zinc ions and provide a binding area for E2 enzyme (Figure 2) [13,14]. RNF168 belongs to RING E3s and has been widely explored as its critical roles in DNA repair. Previous studies have shown that RNF168 plays an important role in the drug resistance of cancer cells and the progression of multiple cancers, on we will elaborate in the following chapters.

## 3. Structure and Functions of RNF168

### 3.1. Structure of RNF168

**Figure 3:**
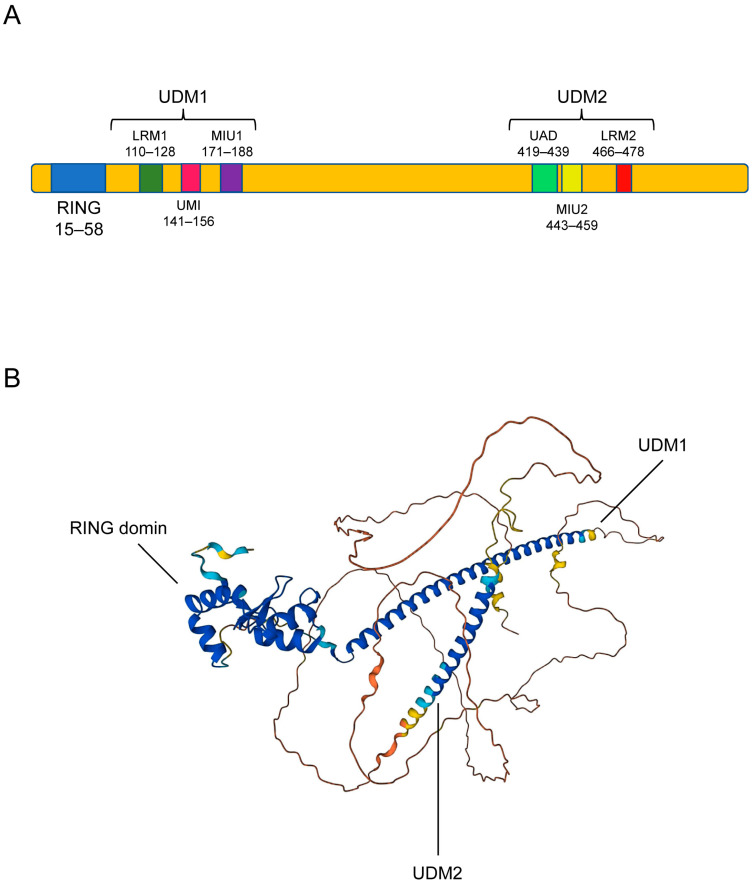
Structure of RNF168. (**A**) RNF168 is composed of a catalytic RING (15–58) domain and two ubiquitin-dependent DSB recruitment modules (UDMs). UDM1 is composed of LRM1 (110–128), UMI (141–156), and MIU1 (171–188), and UDM2 is composed by UAD (419–439), MIU2 (443–459), and LRM2 (466–478). (**B**) RNF168 has three domains, which are RING domain, UDM1 and UDM2 from left to right in the figure. AlphaFold produces a per-residue confidence score (pLDDT) between 0 and 100. Some regions below 50 pLDDT may be unstructured in isolation. Dark blue indicates pLDDT > 90, blue indicates 90 > pLDDT > 70, yellow indicates 70 > pLDDT > 50, and orange indicates pLDDT < 50.

RNF168 is a nuclear protein composed of 571 protein residues, which contains a RING domain at the N terminus, a ubiquitin-dependent double-strand break recruitment module (UDM)1 in the middle, and an UDM2 in the C terminus. The RING domain is the catalytic domain and necessary for RNF168 to complete the function of ubiquitin ligase [4]. UDM1 consists of LR motif 1 (LRM1), UIM- and MIU-related UBD (UMI), and a motif interacting with ubiquitin 1 (MIU1) (Figure 3A). As UDM1 can bind to the K63-linked ubiquitin chain, RNF168 can act as a downstream effector of RNF8 by interacting with the RNF8-ubiquitinated proteins, this process promote recruitment of RNF168 at the DNA breaking site [15]. UDM2 consists of a ubiquitin-associated domain (UAD), an MIU2, and an LRM2 motif. UDM2 facilitates the formation of RNF168 ionizing radiation-induced foci (IRIF), which can amplify ubiquitin signals and recruit downstream DNA repair-related factors [16,17]. In order to better understand the function of RNF168, the 3D structure of RNF168 was found using the AlphaFold Protein Structure Database (AlphaFold DB, https://alphafold.ebi.ac.uk, access on 12 August 2022) and the three domains of RNF168 were marked in Figure 3B [18,19].

### 3.2. Functions of RNF168

#### 3.2.1. DNA Damage Response

DNA double-strand breaks (DSBs) are the most harmful type of DNA damage to cells and RNF168 is a key factor in DSB repair. DNA is a kind of macromolecular polymer composed of deoxynucleotides that store genetic information in organisms. It will cause base damage and mismatching, the covalent cross-linking of DNA strands and DNA single-strand breaks (SSBs), or DSBs when DNA is damaged by exogenous genotoxins and endogenous reactive metabolites [20,21,22]. DNA damage can lead to genetic mutation and proto-oncogene activation, among which DSB is the most harmful type and can cause chromosome aberration [23]. However, DNA damage can be dealt with by a stress mechanism of DNA damage response (DDR), as damaged DNA can activate the phosphatidylinositol-3 kinase-related kinases (PIKK) family protein and trigger a series of downstream reactions [24]; this effect is termed DNA damage repair.

**Figure 4:**
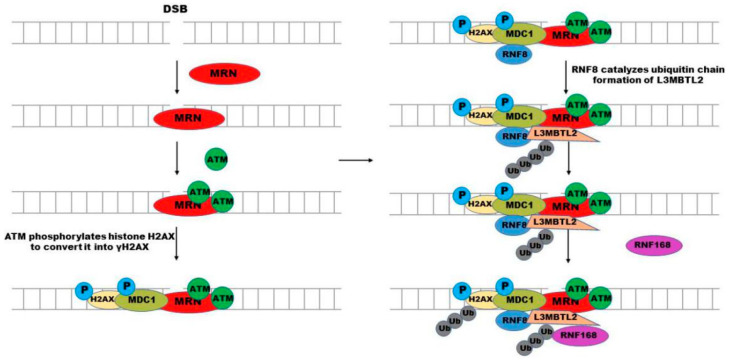
RNF168 participates in the DSB repair process. When the DNA double strand breaks, the MRN complex recognizes the DSB site, and then ATM kinase is recruited to the DSB site by MRN to phosphorylate ser139 of histone H2AX to convert it into γH2AX. MDC1 can sense γH2AX accumulation at the damage site and is then phosphorylated by ATM. RNF8 recognizes the phosphorylated MDC1 and is recruited to the DSB site. ATM-mediated L3MBTL2 phosphorylation promotes its interaction with MDC1 and recruitment to DSB site. Then, RNF8 catalyzes the formation of L3MBTL2 ubiquitin chain, which can promote the recruitment of RNF168 at DSB site. Then, histone N-terminal lysine (k) 13/15 is ubiquitinated by RNF168.

DNA DSB can trigger a series of repair procedures, such as homologous recombination (HR) or non-homologous end connection (NHEJ), and many repair-related proteins work jointly to repair the broken site [25]. This is a cascade reaction, including protein recognition of damage sites, ubiquitination and phosphorylation of histones, and the choice of repair mode will also be contributed by repair-related factors, 53BP1 and BRCA1 [26,27], in which the balance between histone ubiquitination and deubiquitination is particularly critical. RNF168 is one of the key proteins during the ubiquitination process by mediating the recruitment of repair factors at the damage site [28,29,30]. When DNA DSB happens, the DSB damage site will be recognized by the MRE11-RAR50-NBS1 (MRN) complex of DNA damage response network. Then, the ataxia-telangiectasia mutation (ATM) kinase is recruited by MRN to the damage site to phosphorylate the ser139 of histone H2AX and converted it into γH2AX [31]. In addition, the mediator of DNA damage checkpoint 1 (MDC1) can sense γH2AX, and communicate with each other through BRCT domain in MDC1, subsequently accumulating at the damage site where MDC1 is further phosphorylated by ATM [32,33]. The phosphorylated MDC1 can be recognized by RNF8, which recruits RNF168 to this site. Furthermore, ATM-mediated Lethal (3) malignant brain tumor-like protein 2 (L3MBTL2) phosphorylation can also promote its interaction with MDC1 and be recruited to the DSB site. Then, RNF8 directly regulates the formation of K63-linked ubiquitin chain on L3MBTL2, which can facilitate RNF168 recruitment at the damage site, and then N-terminal lysine (K) 13/15 of histones is ubiquitinated by RNF168 (Figure 4) [34,35,36,37]. The ubiquitination of H2A (K) 15 induced by DNA damage can be recognized by 53BP1, in addition, RNF168 can catalyze a K63-linked ubiquitin chain on 53BP1 at K1268, both of them are important for 53BP1 to recruit DNA damage sites [38,39]. Then 53BP1 is recruited to the DNA damage site. 53BP1 is an important protein in DSB reaction, which promotes NHEJ by inhibiting the initial step DNA end resection of HR [38]. Recent studies have shown that histone methyltransferase KMT5A can promote the activity of RNF168 in catalyzing H2A ubiquitination [40]. These two ubiquitin ligases synergistically extend the ubiquitin chain, which recruits other DSB repair-related factors to enhance DSB repair. In addition, the K27-linked ubiquitin chain formed by RNF168 at K13/15 of H2A (X) is required for the proper activation of the DNA damage response and prevents the recruitment of 53BP1 and BRCA1 to the DNA damage site [41].

#### 3.2.2. Other Functions of RNF168

**Figure 5:**
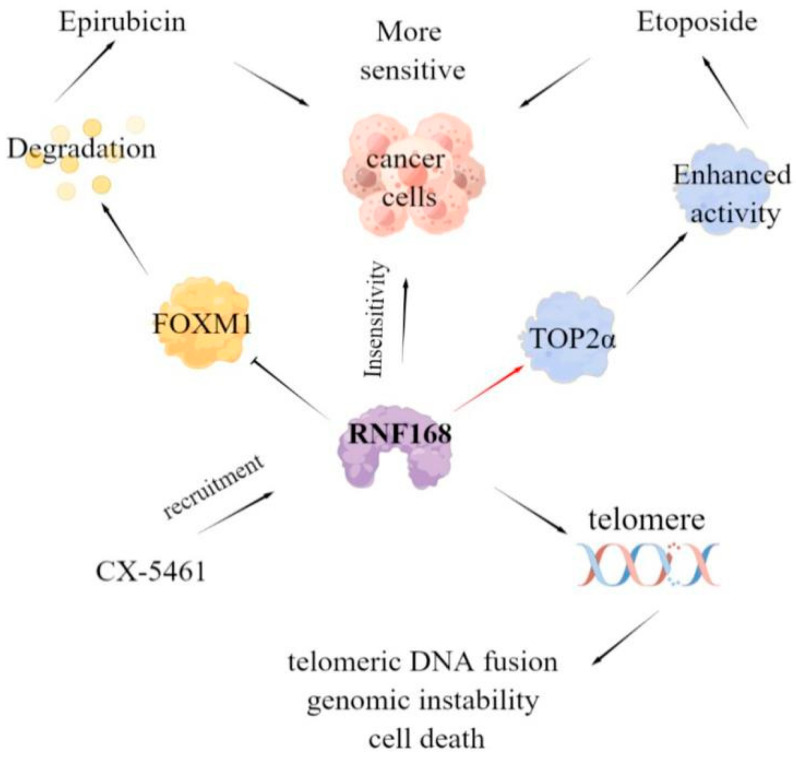
Relationship between RNF168 and tumorigenesis and development. RNF168 can increase the expression of cancer-promoting factors, such as STAT1, WNT3A, β-catenin, and ERα, so it is highly expressed in some cancer cells. RNF168 can promote the degradation of cancer-promoting factors, such as RHOC, or recruit 53BP1 to inhibit HR and cause cell death, so it is lowly expressed in some cancer cells.

In addition, RNF168 also has a certain impact on the efficacy of anticancer drugs. For example, high-level forkhead box M1 (FOXM1) expression can enhance the drug resistance of cancer cells, and studies have shown that RNF168 can promote K48-linked ubiquitination of FOXM1 and reduce the protein level of FOXM1 by promoting its degradation, thus enhancing the sensitivity of breast cancer to Epirubicin [42]. Furthermore, Topoisomerase IIα (TOP2α) is a crucial protein for chromosome condensation and separation and genome integrity [43]. RNF168 can directly interact with TOP2α and mediate its K63 specific polyubiquitination, this polyubiquitination will not lead to protein degradation, but enhance its enzyme activity. Notably, the downregulation of TOP2α expression is one of the mechanisms contributing to drug resistance. Therefore, RNF168 deficiency leads to the resistance of TOP2α catalytic inhibitor ICRF-193 and the cytotoxic anticancer drug etoposide (VP-16) [44]. Moreover, deletion of RNF168 inhibits the chromatin ubiquitin pathway and enhances the sensitivity of cancer cells to the bioavailable derivative of quarfloxin CX-5461 [45]. A recent study showed that a 1,2,3-triazole derivative of quinazoline can directly bind with autophagy-related protein SQSTM1/P62 and E3 ligase RNF168, promote their interaction, and damage the DNA repair mediated by RNF168, thus increasing the sensitivity of colon cancer cell HCT-116 to X-ray radiation. In summary, RNF168 has important value in the drug resistance of tumor cells and the development of anticancer drugs [46]. Based on the function of RNF168 in the DDR pathway, the RNF168-mediated DNA repair pathway would lead to telomeric DNA fusion, genomic instability, and cell death (Figure 5) [47].

#### 3.2.3. Regulation of RNF168 Function

Notably, ubiquitination and deubiquitination will keep relatively stable, which also requires the participation of deubiquitinase (DUB), such as A20/TNFAIP3 of the OTU deubiquitinase family and USP11 or USP3 of the USP protein family [48,49,50]. The RNF168 function in the DDR pathway is regulated by signal pathways and transcription factors in cells, for example, the activation of mTORC1-S6K pathway can phosphorylate the Ser60 site of RNF168, inhibit its E3 ligase activity, and promote RNF168 degradation [51]; and the high expression of the lipolytic inhibitor G0/G1 switch gene 2 (G0S2) can reduce lipid droplet turnover and thereby attenuate RNF168-mediated 53BP1 ubiquitination via activating the mTORC1-S6K signaling and increasing the 53BP1 protein stability in response to ionizing radiation (IR), leading to enhanced DNA repair and glioma radioresistance [52]; bifunctional transcription factor PRMT5 can bind to the promoter of RNF168 and activate RNF168 transcription by regulating histone methylation [53]. Furthermore, the function of RNF168 is also regulated by chemicals, such as Cadmium (Cd) directly binding to RNF168, inducing ubiquitin–proteasome degradation, and suppressing its ubiquitin–ligase activity in vitro [33]. In addition, the RNF168 function can be modulated by viral proteins, such as the BMRF1 protein of Epstein–Barr virus (EBV) inhibiting the recruitment of RNF168 in DSB and histone ubiquitination [54], and human papillomavirus E7 oncoprotein directly binding to RNF168 without affecting its enzymatic activity by targeting a new regulatory domain of RNF168, by which tumor viruses reshape the cellular response to DNA damage [55].

## 4. Roles of RNF168 in Various Cancer Types

### 4.1. High Expression of RNF168 in Cancers

RNF168 plays a key role in DNA damage repair, through which it can affect the occurrence and development of cancer. RNF168 has been found to be highly expressed in various tumor cells, such as breast cancer and prostate cancer, and is closely related to the proliferation, migration, invasion, poor prognosis, and survival rate [31,56,57]. Specifically, RNF168 can participate in cancer progression by regulating the stability of key proteins in the cell signaling pathway and affect the proliferation and invasion of cancer cells.

#### 4.1.1. Breast Cancer

Of all female cancers, breast cancer is the most common cancer with the highest morbidity and mortality, and the proportion may continue to rise [58,59]. Although there have been advances in the treatment of hormone receptor-positive and human epidermal growth factor receptor 2-positive breast cancers, recurrence still always happens [60]. Therefore, there is a critical need to find new molecular targets for breast cancer therapy. RNF168 exhibited a higher level in breast cancers compared with normal breast tissue and correlates with a poor endocrine treatment outcome. Estrogen receptor α (ERα) has been proven to have a main role in breast cancer initiation and proliferation [61]. RNF168 could bind to Erα promoter region and facilitate ERα transcriptional activity, enhance the activity of the ERα signal pathway, and thus promote the proliferation of Erα-positive breast cancer cells [56]. These results indicate that RNF168 plays an important role in the progression of breast cancer. However, whether RNF168 can combine with other targets in breast cancer cells to affect the progress of breast cancer still needs further research.

#### 4.1.2. Esophageal Cancer

Esophageal cancer is one of the most common malignant tumors in China and even throughout the world, and esophageal squamous cell carcinoma has high morbidity and mortality rates [62]. Although more and more therapeutic targets have been identified, the mortality of esophageal cancer is still high. Therefore, it is necessary to understand the molecular mechanism of the occurrence and development of esophageal cancer, identify new biomarkers, and formulate new treatment strategies. RNF168 may stabilize STAT1 protein, which is a core component of the JAK-STAT signaling pathway, by inhibiting the polyubiquitination of STAT1, and thus promote the proliferation and invasion of esophageal cancer cells by activating the JAK-STAT signaling pathway [63,64]. RNF168 expression was also increased in esophageal squamous cell carcinoma (ESCC) compared with normal esophageal epithelium and related to tumor stage and depth of invasion. Mechanistic studies show that RNF168 affects the expression of WNT3A, β-catenin, and glycogen synthase kinase 3β (GSK-3β) [5]. Therefore, RNF168 may participate in the Wnt/β-catenin signaling pathway and thus modulate the proliferation and apoptosis of esophageal cancer cells. However, the role of RNF168 in the pathogenesis of esophageal cancer is still unclear. For example, it needs further research on how RNF168 reduces the polyubiquitination of STAT1 and how it affects the protein of the Wnt/β-catenin signaling pathway to promote the progression of esophageal cancer.

### 4.2. Low Expression of RNF168 in Cancers

#### 4.2.1. BRCA1-Mutant Cancer

In contrast, RNF168 is also lowly expressed in some cancers. Previous studies have found that RNF168 expression is decreased in various BRCA1-mutant cancer cell lines and primary tumors, such as breast cancer and ovarian cancer [30,65]. The recruitment of BRCA1 means cells tend to select HR to repair DNA, which plays an important role in maintaining genomic stability. Therefore, BRCA1 deletion will lead to cell death [66,67]. This is due to the fact that γH2AX ubiquitination mediated by RNF168 at K13/15 can locate 53BP1 to DSB site, and this 53BP1 recruitment will inhibit HR [36,68]. Therefore, BRCA1-deficient cancer cells need to reduce the protein level of RNF168 to maintain a certain HR level in order to ensure survival [65]. Paradoxically, some studies also showed that RNF168 deletion led to the accumulation of R-loop in breast cancer and ovarian cancer cells with BRCA1/2 mutation, and the accumulation of R-loop leads to DSB, senescence and subsequent cell death [69]. Therefore, the role of RNF168 in BRCA1 mutant tumor cells needs further study, such as on RNF168, which may need to reach a certain protein level to serve as an oncogene.

#### 4.2.2. Gastric Cancer

In addition to γH2AX ubiquitination, RNF168 can function through other downstream effectors, among which Ras homolog gene family member C (RHOC), which is a member of the Rho GTPase family [70], is widely explored. Previous studies have shown that RHOC can regulate the invasion and metastasis of cancer cells and promote the progression of breast cancer, pancreatic cancer, lung cancer, ovarian cancer, and cervical cancer [71]. Similarly, RHOC is highly expressed in human gastric cancer tissues and enhances the proliferation, migration, and invasion of cells [72,73,74]. Previous studies have shown that RNF168 is lowly expressed in human gastric cancer tissues and can directly interact with RHOC and promote the degradation of RHOC by increasing the ubiquitination level of RHOC, thus affecting the function of gastric cancer cells [75]. Thus, RNF168 might be used to develop effective therapies for gastric cancer treatment. Whether RNF168 acts as an oncogene or a tumor suppressor is closely related to the target of RNF168 in different cancers. Therefore, more data are needed to determine the function of RNF168 in tumor progression.

#### 4.2.3. Lung Cancer

Similarly, RNF168 was shown to be lowly expressed in lung adenocarcinoma tissues and promote the degradation of RHOC protein by ubiquitinating RHOC, thus suppressing the cancer stem cell (CSC)-like traits of non-small cell lung cancer (NSCLC) cells [57]. Some studies have shown that RNF168 expression is decreased in LKB1-loss-driven NSCLC, which may be due to the fact that depletion of LKB1 leads to an increased RNF168-Ser60 phosphorylation level through activating the mTORC1-S6K pathway. The activated mTORC1-S6K pathway destabilizes RNF168 in a Ser60 phosphorylation-dependent manner, thus promoting genomic instability and participating in the pathogenesis of cancer [51]. The protein level of RNF168 is also regulated by post-translational modification, so the upstream protein that regulates the expression of RNF168 can also be used as a potential target for cancer treatment.

#### 4.2.4. Glioblastoma

**Figure 6:**
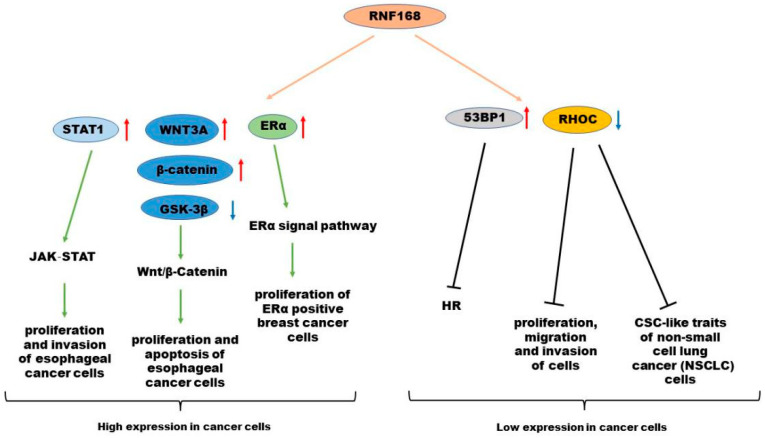
Abnormal expression and clinical significance of RNF168 in various cancer types. RNF168 can increase the expression of cancer-promoting factors, such as STAT1, WNT3A, β-catenin, and ERα. RNF168 can also promote the degradation of cancer-promoting factors, such as RHOC, or recruit 53BP1 to inhibit HR and cause cell death.

A previous report demonstrated that RNF168 expression is suppressed in methylthioadenosine phosphorylase (MTAP)-deficient glioblastoma cells. H2AX plays essential roles in maintaining genome stability and suppressing tumorigenesis. Deletion of MTAP inhibits the expression of PRMT5, the transcriptional activator of RNF168, leading to the loss of RNF168 and hence of H2AX level [53]. In summary, an increasing number of studies about RNF168 indicate that it might be a new target for glioblastoma treatment (Figure 6).

## 5. Future Perspectives

In recent years, a large number of exploration and research studies on RNF168 have led to a new understanding of the role for RNF168 and its regulation mechanism in diseases. As a member of the E3 ubiquitin ligase RING family, RNF168 plays an important role in the occurrence and development of malignant tumor diseases. In this paper, most of the research articles on RNF168 are integrated and systematically sorted, which can help readers quickly become familiar with the research on related topics, and facilitate the retrieval of existing research results, so as to facilitate readers to conduct further research on the role of RNF168 in tumors.

Although many studies have reviewed the relationship between RNF168 and cancer, the underlying mechanisms of RNF168 in cancer or other diseases are still fragmentary. For example, RNF168 is involved in the DDR pathway in cells, while DNA damage repair is involved in many diseases, such as Huntington’s disease (HD) and Parkinson’s disease (PD) [76,77]. Lack of DNA damage repair will result in genomic instability and mutation, the accumulation of which may cause the inactivation of oncogenes or tumor-suppressive genes, subsequently leading to the occurrence of tumors. For example, 50–80% hereditary breast cancer is associated with mutations in BRCA1 or BRCA2 [78,79,80]; most of the genetic mutations are associated with mutations in BRCA2 in pancreatic cancer [81]; the most common mutations in colorectal cancer occur in APC, TP53, and KRAS [82]. On the other hand, deletion of DNA damage repair means the mutation of key genes are unrepaired in a timely manner and the genome is unstable, which may cause cell apoptosis [83]. Notably, under proteasome inhibitor-induced proteotoxic stress, aberrant RNF168-mediated signaling might reflect adaptation to chronic proteotoxic and genotoxic stresses experienced by tumor cells, showing altered DNA repair and responses to genotoxic treatments, genomic instability, and resistance to proteotoxic stress [84].

The above studies are mainly at the basic research stage; it is believed that further research on the structure and function of RNF168 in the future can provide ideas for the development of targeted drugs, especially provide new surface markers, therapeutic targets, and prognostic indicators for tumor treatment, which has important clinical significance. Further research on the role of RNF168 in tumor diseases can provide new ideas for the molecular diagnosis and gene therapy of malignant tumors, facilitate the finding of potential targets for the treatment of malignant tumors, and develop new therapeutic strategies in clinical practice. Additionally, it can facilitate drug discovery efforts using the current platform of reagents and assay readout, targeting all aspects of the ubiquitin cascade and important lessons can be benefit from other classes of RING enzymes. Therefore, RNF168 might be the eligible targets for the development of PROTACs. Additionally, in-depth study of various roles of RNF168 in different diseases may provide new strategies for the targeted treatment of malignant tumors, solve the unsolved problems in the treatment of malignant tumors, and bring progress to medical-related research. Furthermore, understanding the structural characteristics and functions of RNF168 as well as the role mechanism of RNF168 in cancer diseases, especially by establishing transgenic animal models, can better provide a theoretical basis for the clinical treatment of various diseases, especially provide new surface markers, treatment targets, and prognostic indicators for tumor treatment, which is of great clinical significance for the guiding of clinical diagnosis and treatment. Importantly, future work can be carried out to explore RNF168 inhibitor or activator, which may facilitate the development of RNF168-targeted therapy for tumors or other related diseases.

## 6. Conclusions

The present review exposed the structure and function of E3 ubiquitin ligase RNF168 and its relationship with cancer, especially the role of RNF168 in carcinogenesis and development. RNF168 can participate in the occurrence of cancer through a variety of ways and is closely related to the drug resistance of cancer cells. Therefore, RNF168 has the potential as an anticancer target.

## Data Availability

Not applicable.

## Abbreviations

RNF168	RING finger protein 168
PTM	Post-translational modification
PROTAC	Proteolysis-targeting Chimeras
HECT	Homologous to E6AP C-terminals
RBR	RING between RING
UDM	Ubiquitin-dependent double-strand break recruitment module
LRM	LR motif
UMI	UIM- and MIU-related UBD
MIU	Motif interacting with ubiquitin
UAD	Ubiquitin-associated domain
IRIF	Ionizing radiation-induced foci
DSB	DNA double-strand break
SSB	DNA single-strand break
ROS	Reactive oxygen species
DDR	DNA damage response
PIKK	Phosphatidylinositol-3 kinase-related kinases
HR	Homologous recombination (HR)
NHEJ	Non-homologous end connection
ATM	Ataxia-telangiectasia mutation
MDC1	Mediator of DNA damage checkpoint 1
L3MBTL2	Lethal (3) malignant brain tumor like protein 2
FOXM1	Forkhead box M1
TOP2α	Topoisomerase IIα
IR	Ionizing radiation
EBV	Epstein Barr virus
ERα	Estrogen receptor α
ESCC	Esophageal squamous cell carcinoma
GSK-3β	Glycogen synthase kinase 3β
RHOC	Ras homolog gene family member C
CSC	Cancer stem cell
NSCLC	Non-small cell lung cancer
MTAP	Methylthioadenosine phosphorylase
HD	Huntington’s disease
PD	Parkinson’s disease
